# “Playing on” or “calling time”? retention and motivation for sport officials

**DOI:** 10.3389/fspor.2026.1789166

**Published:** 2026-04-13

**Authors:** Aden Kittel, Ian Cunningham, Joshua Adie, Stirling Sharpe, Paul Larkin

**Affiliations:** 1 Institute of Health and Sport, College of Health and Biomedicine, Victoria University, Melbourne, VIC, Australia; 2 Centre for Sport Research within Institute for Physical Activity and Nutrition, Deakin University School of Exercise and Nutrition Sciences, Burwood, VIC, Australia; 3 School of Applied Sciences, Edinburgh Napier University School of Applied Sciences, Edinburgh, United Kingdom; 4 School of Health, University of the Sunshine Coast, Sippy Downs, QLD, Australia; 5 Discipline of Sport and Exercise Science, University of Canberra, Canberra, ACT, Australia; 6 MSA Research Centre, Maribyrnong Sports Academy, Melbourne, VIC, Australia

**Keywords:** development, motivation, officials, referee, umpires

## Abstract

**Introduction:**

Attrition is a key issue in sports officiating and has rightfully received extensive attention in the literature highlighting issues such as abuse and lack of perceived organizational support. However, few studies in comparison understand the reasons for officials to stay in their role (i.e., retention) and underlying motivation.

**Methods:**

Current officials were recruited (*n* = 293) from multiple countries and sports (mainly soccer, rugby union, cricket, hockey) to complete an online questionnaire, focusing on sport motivation and reasons to continue officiating.

**Results:**

Based on the Sport Motivation II scale (SMS-II), officials report high Intrinsic and Identified modes of regulation. There were no differences between genders in any of the modes of regulation in SMS-II. Those that intend to continue reported higher levels of Intrinsic, Integrated and Identified than those that were unsure of continuing. Amotivation was significantly higher for those that were unsure of continuing and did not intend to continue, than those who did intend to continue. Results indicated professional level and soccer officials appear to exhibit higher levels of motivation. Personal Drive and Personal Challenge were the most prominent reasons to continue, whereas Money was the least selected reason.

**Discussion:**

This study provides important implications for officiating managers in the retention profiles of active officials, depending on various factors.

## Introduction

Sports officials play a vital yet often underappreciated role in the sporting ecosystem. Despite their importance to sporting competitions being delivered effectively across all performance levels (i.e., grassroots to elite international competition), a shortage of available officials is frequently reported (40). Continued high attrition rates indicate the retention of officials is a critical sport management challenge (1, 2), wherein this issue is not merely isolated to specific sports in certain geographic areas, but rather a pervasive global issue for all sports and regions (3). While performance factors such as physiology, decision making, and bias have been widely explored in officiating research due to their impact on in-game performance, there has been comparatively less focus on development and retention factors (4). Given this, it is important for the officiating literature and industry to better understand development and retention factors, as this will aid the industry in addressing the key issues of retaining sport officials.

A common objective for sport governing bodies is to increase their participation rates, yet the challenge for growing a participation base is the need to recruit and retain enough match officials to facilitate the increased number of matches (5). Retaining officials is more cost effective than recruiting and training new officials, yet many officials will leave their role within the first five years (6). Therefore, the retention of officials should be a strategic imperative for sport organisations. There are some strategies recommended to be effective in retaining officials such as developing a sense of community (3), access to quality mentors (7), training opportunities (41), and appropriate recognition (8). However, these studies do not take into account the initial motivations of the match officials in their samples and as such these strategies may not reach the psychological needs or motivations of all officials.

There are a range of factors why sport officials leave their role (i.e., attrition) (9–11). For example, verbal and physical abuse has been well-documented as a challenge experienced by sport officials and can lead to them leaving their role [see Mojtahedi et al. (12) for a review]. Other factors that can lead to officials' attrition include, low perceived organisational support (13, 14), role satisfaction (42), self-efficacy (43), quality of officials' social supports and communities (44), administrative considerations and inequity (3), and officials' negative social experiences (15, 16). A negative experience for one of these factors can unfortunately lead to a downward trend in well-being and mental health outcomes (9). These findings provide valuable insights for sport officials’ developers to avoid attrition, by developing environments that limit these factors.

### The role of motivation in predicting sports officials' intentions to continue

Although there has been considerable research into why sports officials leave, it's equally important to explore the factors that contribute to their retention (i.e., why officials choose to stay and continue in the role). For example, studies have ascertained that negative social environments can be a deterring factor for officials to continue, particularly female officials (15, 16). Despite these negative environments, when female officials are retained in their sport, this is often due to their intrinsic enjoyment and staying involved with the sport, plus the benefits of being a part of a community (3, 17, 18). Sense of community and social support are seen as important predictors of retention because they are integral to the interpersonal networks in which officials' participation occurs (44). It has been suggested that retention strategies for officials should concentrate on early career officials (less than five years' experience) as this group is likely to be most impacted by attrition causing factors (5). Furthermore, it has been reported that officials have a strong desire to belong and be retained in their role (19).

A sport psychology perspective can be useful for academics and practitioners to understand the motivations for sport officials to continue in their role. Regardless of sport, it has been identified that sport officials continue for both intrinsic motivations and social reasons (8). Self-determination theory (SDT) suggests motivation occurs on a continuum from amotivation (i.e., reduced motives to persist in goal-directed behaviour) to extrinsic motivation towards more intrinsic sources of motivation (20). Shifting towards more intrinsic sources of motivation involves satisfising basic psychological needs of competence, autonomy, and relatedness to others in each task (20). Autonomy refers to feeling in control of one's actions and choices that align with personal values and priorities. Competence refers to feeling effective at a task and experiencing a sense of mastery. Relatedness refers to a feeling of social connection to others and a sense of group belonging. Early research that integrated SDT to understand motivation factors contributing to retention of sport officials found perceived relatedness predicted a track and field officials' likelihood to continue. In a study that explored female sport officials' psychological needs of self-determination and reasons related to retention, key relationships were discovered as an important retention function (18). Greater feelings of relatedness were associated with mentorship support, renumeration, and feelings of autonomy and volition, while lack of stress and continuing education were associated with most factors of self-determination (18). These findings highlight the importance of understanding sport officials' motivations through an SDT lens. Therefore, further research is required to develop tailored recruitment strategies that are sport and context-specific, addressing the motivational factors that emerge and contribute to officials' intentions to remain involved and continue.

While some research has begun to explore sport official motivation factors in relation to retention, these studies have been limited by focusing on one gender (18), one geographical region (8, 21) or one sport (22). Although these studies have provided unique and valuable insights to the literature, the present study looks to build on previous research by exploring these retention factors and motivations through an international perspective across different sports, ages, and genders. Further, perceived organisational support has been identified as a predictor, but limited research explores the underlying motivations. This knowledge would help the sporting industry in their ongoing challenge of retaining sport officials. Such knowledge may also contribute to targeted recruitment strategies considering officials' intentions to remain involved and continue. Therefore, we sought to answer the following research questions:

1. What are the motivation profiles of officials differing by gender, intention to continue, sport, and current officiating level?
2. What are the key reasons to remain involved as a sports official?
3. How do these key reasons differ by intention to continue, gender, sport, and current level?

## Methods

### Participants

A total of 369 sports officials, from 44 different countries, participated in the study on a voluntary basis. All participants were actively engaged in officiating their respective sports at the time of the project. The sample was comprised of 308 males; 47 females; and 14 participants who identified as other. The majority of the participants were from four countries, Australia (*n* = 103); the USA (*n* = 65); the United Kingdom (*n* = 54); and Canada (*n* = 21), accounting for 66% of the total sample. Of these 369 sports officials, 66 failed to complete the entire SMS-II portion of the questionnaire and were subsequently removed from analysis resulting in a sample of 303 sports officials (female = 38, male = 255, othe*r* = 10). Due to insufficient data points, only data from female and male participants were included in the analyses (total *n* = 293). Participants were across the following predefined age categories: 13–15 = 2; 16–18 = 4; 19–24 = 29; 25–30 = 29; 31–40 = 56; 41–50 = 67; 51+ = 106. Ethics approval was granted by the lead researcher's institutional human research ethics committee (Victoria University Human Research Ethics Committee; HREC22-08). Informed consent was obtained from all participants prior to the data collection period.

### Officiating recruitment survey

This study forms part of a larger project investigating officials' entry and recruitment, their motivations to continue, and their perceptions of the talent development environment. To explore this, a survey was developed and incorporated five main sections, (i) demographics; (ii) sports officiating entry and recruitment; (iii) sports officiating intention to continue; (iv) sports officiating talent environment; and (v) sport motivation scale. The focus of the current study is section three (i.e., sports officiating intention to continue) and five (i.e., sport motivation scale) and the demographic information. The demographic section included questions about the participants' gender; age; country; sport; and level at which they currently officiate. The sports officiating intention to continue section asked participants “What has motivated you to stay involved in officiating (tick all that apply)?” These options include *Personal Drive, Personal Challenge, Get to the highest level (a career path), Money, To give back, Remain involved with the officiating community, and Mentor younger officials*. The survey was informed by previous research on common motivational factors in officiating, including intrinsic and extrinsic motivators (13) and social and sport-related reasons Hancock et al. (8). Additionally, other theoretical frameworks such as Self-Determination Theory [SDT; see Sunde et al. (18) and sport government-developed tools (23)], were integrated to inform the question development. To refine the survey for an applied perspective, consultation was held with key sports officiating stakeholders, including industry experts from governing body officiating managers in Australia and leading sports officiating researchers.

To measure the participants' motivation, the Sport Motivation Scale II (SMS-II) (24) was used. This scale is a modified version of the original SMS scale (25), and consists of 18 items distributed across six subscales: non-regulation (i.e., amotivation); external regulation; introjected regulation; identified regulation; integrated regulation; and intrinsic regulation. Each item is evaluated using a seven-point Likert scale ranging from 1 (does not correspond at all) to 7 (corresponds completely), where higher scores indicate greater motivation. Overall, the SMS-II aligns closely with self-determination theory (20).

### Procedure

A purposeful convenience sampling approach was used in this study. Following the development of the questionnaire, the research team disseminated the link to the online survey through multiple channels, including, personal networks; sporting organisations’ mailing lists; and social media posts on popular sport officiating pages. This sampling strategy was aimed at communities with a strong focus on sports officiating. Interested participants could then access the survey by following the link provided either on the email or social media post. Once the participant clicked the link, they were taken to the participant information sheet and asked to provide informed consent prior to commencing the survey. Participants were informed their involvement was completely voluntary and their responses would remain anonymous. After providing consent, participants were taken directly to the survey which took approximately 20 min to complete.

### Statistical analysis

All subscales of the SMS-II violated assumptions of normality, therefore a series of Kruskal–Wallis tests (45) were conducted to examine differences in SMS-II subscales across gender (female = 38; male = 255), intention to continue officiating (yes = 255; maybe = 24; no = 14), sport officiated (socce*r* = 95; cricket = 66; rugby = 44; hockey = 45), and current level of officiating (professional = 28; semi-professional = 94; talent pathway = 36; local/grassroots = 131). Note; 4 participants did not respond and were removed for comparisons relating to this variable). Effect sizes were calculated using eta-squared (*η^2^_H_*), with values of < 0.06 (small); 0.06–0.13 (medium); and > 0.13 (large) used for interpretation (46, 47). Significant results were followed by pairwise Dunn's tests with Bonferroni corrections for multiple comparisons.

To explore participants' reasons and motivations for continuing as an official (participants were able to select multiple), descriptive statistics relating to the percentage of participants who selected each reason were provided. Inferential chi-squared tests were not performed in this case, as there was no theoretical justification for specified expected frequencies for each option. Chi-squared tests of independence were conducted to explore differences relating to gender, intention to continue officiating, and current level of officiating. During the review process it was requested that we also explore differences in reasons relating to sport and age group. Chi-squared tests only proceeded when fewer than 20% of cells contained expected frequences <5, and no cells contained expected frequencies <1. For the sport variable, chi-squared tests were performed only on data for the four most frequent sports (soccer, cricket, rugby, hockey; *n* = 250). Age was explored descriptively as the data did not meet the minimum requirements relating to expected frequencies. All significant chi-squared results were followed by analysis of adjusted standardised residuals (48). Given that adjusted standardised residuals are *z*-scores, absolute values were interpreted as significant at *p* *<* 0.05 (*z* > 1.96), *p* *<* 0.01 (*z* > 2.58), and *p* < 0.001 (*z* > 3.29). All analyses were conducted in SPSS version 29.

## Results

### Motivation profile as measured by SMS-II

There were no significant gender differences (see Table 1) in intrinsic regulation (*χ^2^(1)* = 0.62, *p* = .431, *η*^2^_H_ = .00), integrated regulation [*χ^2^(1)* = 0.88, *p* = .349, *η*^2^_H_ = .00], identified regulation [*χ^2^(1)* = 0.03, *p* = .869, *η*^2^_H_ = .00], introjected regulation [*χ^2^(1)* = 0.02, *p* = .881, *η*^2^_H_ = .00], external regulation [*χ^2^(1)* = 1.60, *p* = .206, *η*^2^_H_ = .002], or non-regulation [*χ^2^(1)* = 3.38, *p* = .066, *η*^2^_H_ = .008]. All gender differences represented a negligible effect size (*η^2^_H_* < .01).

**Table 1:** The mean and standard deviation of the SMS-II subscales by gender.

Subscale	Female (*n* = 38)	Male (*n* = 255)
Intrinsic	5.31 (1.25)	5.46 (1.19)
Integrated	5.04 (1.28)	4.72 (1.58)
Identified	5.34 (1.35)	5.25 (1.50)
Introjected	4.03 (1.52)	4.01 (1.46)
External	2.79 (1.49)	2.51 (1.46)
Amotivation	2.89 (1.81)	2.31 (1.40)

There were also no significant differences relating to intention to continue for introjected regulation [*χ^2^(2)* = .93, *p* = .627, *η*^2^*_H_* = .000] or external regulation [*χ^2^(2)* = 3.91, *p* = .142, *η*^2^*_H_* = .007] (see Table 2) representing negligible effect sizes. There were however significant differences associated with the officials' intention to continue including small effects for intrinsic regulation [*χ^2^(2)* = 11.49, *p* = .003, *η^2^*_H_ = .033], integrated regulation [*χ^2^(2)* = 10.68, *p* = .005, *η^2^_H_* = .03], and identified regulation [*χ^2^(2)* = 13.59, *p* = .001, *η^2^_H_* = .04] while non-regulation [*χ^2^(2)* = 33.72, *p* < .001, *η^2^_H_* = .11] represented a significant medium effect.

**Table 2:** The mean and standard deviation of the SMS-II subscales by participants’ intention to continue officiatin*g.*

Subscale	Yes (*n* = 255)	Maybe (*n* = 24)	No (*n* = 14)
Intrinsic	5.54 (1.12)	4.79 (1.40)*	4.69 (1.54)
Integrated	4.88 (1.48)	3.79 (1.52)**	4.31 (2.11)
Identified	5.38 (1.41)	4.29 (1.49)**	4.62 (1.97)
Introjected	4.04 (1.42)	3.81 (1.72)	3.79 (1.87)
External	2.59 (1.44)	2.17 (1.48)	2.41 (1.78)
Amotivation	2.17 (1.28)	3.31 (1.55)***	4.69 (1.88)**

* *p* < .05, **p < .01, ***p < .001 compared to those who intend to continue.

Post-hoc Dunn's tests revealed that participants who intended to continue had significantly higher scores compared to those who were unsure for intrinsic regulation (*z* = 2.68, *p* = .022), integrated regulation (*z* = 3.17, *p* = .005), and identified regulation (*z* = 3.50, *p* = .001), and were significantly lower on non-regulation (i.e., Amotivation; *z* = −3.69, *p* = .001). Officials who intended to continue had significantly lower scores for non-regulation compared to those who did not intend to continue (*z* *=* −4.72, *p* < .001). All other pairwise comparisons relating to intention to continue were non-significant.

There were no significant differences relating to current level of officiating (see Table 3) for introjected motivation [*χ*^2^(3) = 6.51, *p* = .089, *η*^2^ = .012] and amotivation [*χ*^2^(3) = 2.79, *p* = .424, *η*^2^ = .00]. There was however significant differences relating to current level including small effects for intrinsic motivation [*χ*^2^(3) = 8.65, *p* = .034, *η*^2^ = .02], identified motivation [*χ*^2^ (3) = 9.32, *p* = 0.025, *η*^2^ = 0.02], external motivation [*χ*^2^ (3) = 8.62, *p* = .035, *η*^2^ = .02], and a medium effect for integrated motivation [*χ*^2^ (3) = 20.46, *p* < .001, *η*^2^ = 0.061].

**Table 3:** The mean and standard deviation of the SMS-II subscales by current level of officiating.

Subscale	Professional (*n* = 28)	Semi-professional (*n* = 94)	Talent Pathway (*n* = 36)	Local/Grassroots (*n* = 131)
Intrinsic	5.99 (0.75)	5.51 (1.22)	5.45 (0.96)	5.26 (1.28)
Integrated	5.62 (1.27)	5.06 (1.50)	4.57 (1.21)	4.41 (1.62)
Identified	5.82 (1.32)	5.37 (1.50)	5.39 (1.26)	5.03 (1.53)
Introjected	4.32 (1.76)	4.23 (1.44)	3.78 (1.28)	3.83 (1.47)
External	2.88 (1.70)	2.86 (1.57)	2.44 (1.21)	2.31 (1.36)
Amotivation	1.98 (1.01)	2.63 (1.66)	2.20 (1.06)	2.32 (1.47)

Post-hoc Dunn's tests revealed that professional officials had significantly higher intrinsic motivation (*z* = 2.78, *p* = .032), integrated motivation (*z* = 3.83, *p* < .001), and identified motivation (*z* = 2.82, *p* = .029) compared to grassroots officials. Professional officials also had significantly higher integrated motivation compared to talent pathway officials (*z* = 3.02, *p* = .015). Semi-professional officials had significantly higher integrated motivation (*z* = 3.07, *p* = .013), and external motivation (*z* = 2.73, *p* = .038) compared to grassroots officials. All other comparisons relating to current level were non-significant.

There were no significant differences relating to sport officiated (see Table 4) for external motivation [*χ*^2^(3) = 4.55, *p* = .208, *η*^2^_H_ = .006] and amotivation [*χ*^2^(3) = 1.80, *p* = .615, *η*^2^_H_ = .000].

**Table 4:** The mean and standard deviation of the SMS-II subscales by sport officiated.

Subscale	Soccer (*n* = 95)	Cricket (*n* = 66)	Hockey (*n* = 45)	Rugby (*n* = 44)
Intrinsic	5.76 (1.09)	5.24 (1.27)	5.40 (1.24)	4.96 (1.23)
Integrated	5.01 (1.65)	4.38 (1.70)	4.84 (1.41)	4.37 (1.22)
Identified	5.58 (1.40)	4.92 (1.66)	5.40 (1.32)	4.81 (1.39)
Introjected	4.20 (1.57)	3.53 (1.45)	4.22 (1.31)	3.74 (1.27)
External	2.75 (1.55)	2.38 (1.53)	2.36 (1.30)	2.30 (1.19)
Amotivation	2.45 (1.42)	2.28 (1.52)	2.39 (1.42)	2.45 (1.42)

There was however significant differences relating to current level including small effects for intrinsic motivation [*χ*^2^(3) = 16.64, *p* < .001, *η*^2^_H_ = .055], integrated motivation [*χ*^2^(3) = 10.18, *p* = .017, *η*^2^_H_ = .029], identified motivation [*χ*^2^(3) = 13.34, *p* = .004, *η*^2^_H_ = .042], introjected motivation [*χ*^2^(3) = 11.36, *p* = .010, *η*^2^_H_ = .034].

Post-hoc Dunn's tests revealed that soccer officials had significantly higher intrinsic motivation (*z* = 3.80, *p* < .001), integrated motivation (*z* = 2.65, *p* = .049), and identified motivation (*z* = 3.23, *p* = .007) compared to rugby officials. Soccer officials also had significantly higher intrinsic motivation (*z* = 2.76, *p* = .035), identified motivation (*z* = 2.65, *p* = .048), and introjected motivation (*z* = 2.95, *p* = .019) compared to cricket officials.

### Reasons for continuing as an official

The most frequently reported reason for continuing as an official was *Personal challenge* (65.2% of respondents selecting this option) followed by *Personal drive* (59.7%); *Remain involved* (54.3%); *To give back* (50.5%); *Mentor younger officials* (50.2%); and *Get to the highest level (a career path)* (42%). The least frequently reported selection was *Money* (24.9%).

A chi-squared test of independence revealed female officials (*observed* = 65.8%) were significantly more likely to select *Mentor younger officials* compared to male officials (*observed* = 47.84%) representing a small effect, *χ*^2^(1)= 4.26, *p* = .039, *Cramer's V* = 0.121. No other significant gender differences were observed for the remaining motives to continue as an official (see Table 5).

**Table 5:** Reasons for continuing officiating. Reported as % of total respondents (*z*-score).

	Personal Challenge	Personal Drive	Remain involved in the community	To give back	Mentor younger officials	Reach the highest level (career path)	Money
Overall (*n* = 293)							
	65.2%	59.7%	54.3%	50.5%	50.2%	42.0%	24.9%
Gender							
Female (*n* = 38)	68.4% (0.45)	50% (−1.31)	52.6% (−0.22)	47.4% (−0.42)	65.8% (2.06)*	52.6% (1.43)	23.7% (−0.19)
Male (*n* = 255)	64.7% (−0.45)	61.2% (1.31)	54.5% (0.22)	51% (0.42)	47.8% (−2.06)*	40.4% (−1.43)	25.1% (0.19)
Intention to continue							
Yes (*n* = 255)	67.5% (2.11)*	62.4% (2.37)	55.7% (1.61)	50.6% (0.07)	51.4% (1.07)	42.7% (0.69)	23.9% (−1.02)
Maybe (*n* = 24)	58.3% (−0.74)	41.7% (−1.88)	41.7% (−1.29)	50% (−0.05)	33.3% (−1.72)	25% (−1.76)	25% (0.01)
No (*n* = 14)	35.7% (−2.37)*	42.9% (−1.32)	42.9% (−0.88)	50% (−0.04)	57.1% (0.53)	57.1% (1.18)	42.9% (1.59)
Current level							
Professional (*n* = 28)	82.1% (1.96)	92.9% (3.75)***	71.4% (1.87)	53.6% (0.38)	46.4% (−0.42)	82.1% (4.50)***	35.7% (1.44)
Semi-Professional (*n* = 94)	64.9% (−0.13)	54.3% (−1.35)	50% (−1.11)	39.4% (−2.55)	51.1% (0.21)	57.4% (3.64)***	27.7% (0.85)
Talent Pathway (*n* = 36)	52.8% (−1.70)	63.9% (0.53)	66.7% (1.55)	63.9 (1.76)	63.9% (1.76)	36.1% (−0.79)	27.8% (0.48)
Grassroots (*n* = 131)	65.6% (0.08)	55.7% (−1.31)	51.1% (−1.10)	53.4% (1.01)	46.6% (−1.12)	24.4% (−5.57)***	19.1% (−1.97)
Sport (*n* = 250)							
Soccer (*n* = 95)	66.3% (−0.23)	54.7% (−1.02)	49.5% (−1.02)	42.1% (−1.76)	50.5% (0.63)	49.5% (1.57)	31.6% (3.00)**
Cricket (*n* = 66)	60.6% (−1.33)	51.5% (−1.40)	51.5% (−0.40)	50% (0.15)	39.4% (−1.63)	37.9% (−1.02)	10.6% (−2.53)*
Rugby (*n* = 44)	75.6% (1.32)	75.6% (2.52)	64.4% (1.61)	60% (1.60)	44.4% (−0.53)	42.2% (−0.15)	6.7% (−2.69)**
Hockey (*n* = 45)	70.5% (0.51)	61.4% (0.38)	54.5% (0.14)	52.3% (0.45)	59.1% (1.62)	38.6% (−0.67)	31.8% (1.81)
Age							
13–15 (*n* = 2)	100%	100%	0%	100%	50%	50%	100%
16–18 (*n* = 4)	25%	25%	0%	75%	75%	50%	0%
19–24 (*n* = 29)	69%	69%	59%	17%	21%	83%	31%
25–30 (*n* = 29)	66%	79%	38%	34%	31%	72%	31%
31–40 (*n* = 56)	66%	66%	54%	38%	57%	55%	32%
41–50 (*n* = 67)	58%	67%	48%	61%	57%	25%	31%
50+ (*n* = 106)	54%	59%	60%	62%	55%	25%	13%

* *p* *<* *.05,* **p *<* *.01,* ***p *<* *.001.*

Age-group data is only presented descriptively as did not meet minimum requirements of expected frequencies.

A series of chi-squared tests of independence revealed there were significant differences in response frequencies relative to an officials' current level of officiating. A large significant association was found for *Get to the highest level (a career path)*, *χ*^2^(3) = 44.78 *p* < .001, *Cramer's V* = 0.39. Standardised residuals indicated professional (*observed* = 82.1%, *z* = 4.5) and semi-professional officials (*observed* = 57.4%, *z* = 3.64) selected this reason more frequently than expected, whereas grassroots officials (*observed* = 24.4%, *z* = −5.57) selected it less frequently than expected. A moderate significant difference was also identified for *Personal drive*, *χ*^2^(3) = 15.09, *p* = .002, *Cramer's V* = 0.23. Standardised residuals showed professional officials (*observed* = 92.8%, *z* = 3.75) were more likely to select this option than expected. There were no significant differences in response frequencies with respect to current level for the remaining reasons to continue (see Table 5).

Officials' intention to continue officiating was significantly associated with selecting *Personal drive*, *χ*^2^(2) = 6.43, *p* = .040, *Cramer's V* = 0.15. Standardised residuals revealed that officials intending to continue (*observed* = 67.5%, *z* = 2.11) were more likely to choose *Personal challenge*, whereas those officials not intending to continue (*observed* = 35.7%, *z* = −2.37) were less likely than expected to do so. No significant associations were observed between intention to continue and the remaining reasons (see Table 5).

There was also a moderate association between the officials' primary sport and selecting *Money*, *χ*^2^(3) = 18.94, *p* < .001, *Cramer's V* = 0.28. Standardised residuals indicated soccer referees (*observed* = 31.6%, *z* = 3.00) chose this option more frequently than expected, whereas cricket (*observed* = 10.6%, *z* = −2.53) and hockey (*observed* = *6*.7%, *z* = −2.69) officials chose this option less frequently than expected. There were no other differences relating to primary sport for the remaining motives (see Table 5).

## Discussion

This study examined how motivations differ among officials based on their gender, intention to continue, sport, and current performance level. Additionally, it examined the reasons officials chose to remain in officiating, and whether these reasons vary by the same factors. No differences in motivation were identified in gender-based comparisons. However, officials who intended to continue reported significantly higher intrinsic, integrated, and identified motivation compared to those uncertain about their future in officiating. Conversely, those who intended to continue exhibited significantly lower levels of amotivation than those uncertain or planning to leave the role. When comparing competitive level, the officials were currently performing at, key differences emerged across intrinsic, integrated, identified motivation, with professional officials typically higher on these factors. When comparing by sport, soccer officials reported higher levels of intrinsic and identified motivation than both cricket and hockey officials, and higher integrated compared to cricket, and higher introjected compared to hockey. The most frequently cited reasons for continuing officiating among the entire sample were *Personal Drive* and *Personal Challenge*, while *Money* and *Reach the Highest level* were the least selected reasons. Female officials were more like to select *Mentor Younger Officials* than their male counterparts. Those that intended to continue were more likely to select *Personal Challenge*. However, *Reach the Highest Level* was more commonly selected by elite and sub-elite officials than by community-level officials, and professional level officials were more likely to select *Personal Drive*. Rather than reflecting diminished motivation among grassroots officials, this pattern may indicate differences in perceived progression opportunities within officiating pathways. Community-level officials may remain highly motivated to officiate while simultaneously perceiving limited feasibility of long-term advancement, particularly where development structures are less visible or accessible.

Officials appear to exhibit high levels of motivation, particularly intrinsic reasons, consistent with previous research on officiating motivation (8, 18, 26). Differences in motivation emerged when comparing officials who intended to continue with those uncertain about their future in officiating roles. Specifically, officials who selected “yes” reported higher levels of intrinsic, integrated and identified modes of regulation, alongside lower levels of amotivation. This suggests that continuing in officiating roles is associated with greater enjoyment, alignment with personal values, and a stronger perception of the role's importance (24). Although the present study did not directly assess causes of motivational decline, self-determination theory suggests that loss of motivation may arise when officials experience frustration of autonomy, competence, or relatedness. For example, limited input into progression decisions, insufficient developmental feedback, or social isolation within officiating groups may undermine internalisation and increase amotivation. These mechanisms were not measured directly but offer a plausible explanation for uncertainty about continued involvement. Notably, *Identified Regulation* (i.e., personal importance) was one of the highest rated motivational factors, offering a new perspective on officiating attrition research, which has highlighted lack of recognition as a key reason for attrition for certain types of officials (8). By comparing officials with different intentions to continue, this study builds on existing research on sport officiating retention (8, 18, 21) and contributes to a deeper understanding of the motivational factors influencing officials' decisions to remain in the profession. Practically, the findings highlight that motivational profiles may be relevant not only for retention but also for progression within officiating pathways and may inform how governing bodies structure progression pathways.

The current findings build on those of Livingston and Forbes (21) and Symonds and Russell (27), who also explored types of motivation postulated by SDT and its' various modes of regulation, highlighting key regulatory factors associated with retention. No significant gender differences were found in reported motivation levels, which contrasts with previous research indicating that females exhibit higher amotivation, and males higher intrinsic motivation (21), while Symonds and Russell (27) showed male officials display higher integrated regulation, introjected regulation, and amotivation compared to female officials. Including our study, sample characteristics, such as officiating context and cultural setting could influence gender differences that require further investigation. However, it is also plausible that broader structural developments within officiating, including increased professionalisation and targeted developmental support for female officials, have contributed to more comparable motivational experiences across genders. From a self-determination theory perspective, motivational quality may be shaped more by environmental support and need satisfaction than by gender *per se*. This discrepancy may be attributed to the sampling approach for the current study, which may have included participants who were more intrinsically motivated overall.

Key differences emerged when analysing performance levels, with elite officials reporting higher scores on three key regulation factors towards the intrinsic end of the motivation spectrum (24), including intrinsic motivation (higher than community-level officials), integrated motivation (higher than talent pathway officials), and identified regulation (higher than community-level officials). A similar trend occurred where soccer officials tended to score higher than hockey officials in intrinsic, identified and integrated motivation, plus higher than cricket officials in intrinsic, identified and introjected motivation. To our knowledge, no previous studies have examined these motivational factors across different sports or officiating performance levels. As such, our research could give a higher degree of confidence to practitioners that the identified regulation results are generalisable across sports and performance levels. From a practical perspective, these differences suggest that progression to elite levels may be associated with, or supported by, increasingly internalised forms of motivation. If elite officials experience stronger enjoyment, identity alignment, and personal endorsement of their role, development systems may need to create opportunities for these qualities within the pathway. This may be achieved by governing bodies embedding autonomy-supportive coaching (28) and reflective practice opportunities (29) within development pathways (30). Demotivation may also emerge at key transitional points within an officiating career. Officiating careers are characterised by recurring change-events, such as promotion, deselection, role transitions, and performance errors, which can disrupt stability and require psychological adaptation (31). Periods of stalled progression, unmet expectations, or reduced appointment frequency may therefore represent moments of motivational vulnerability, particularly when progression pathways lack clarity or support.

The most prominent reasons for continuing as a sports official were *Personal Drive* and *Personal Challenge*, while *Money* was the least selected reason. This aligns with previous research emphasising the role of intrinsic motivation for sustained involvement in officiating (8) and other research that shows monetary incentives are less influential reasons to be involved (11, 17). No significant differences were found in reasons for continuing when comparing gender or sports. The absence of gender-based differences is particularly noteworthy, given the growing call for more research on female officials (18, 32, 33). In particular, it is interesting to note there were no significant gender-based differences in relation to remaining involved in the community, as past research has highlighted this as a significant concern in retaining female officials (18, 33, 34). This may indicate a stronger sense of relatedness (a key component of self-determination theory) among female officials. The primary differences observed were related career progression, with elite and sub-elite officials more likely to cite attaining higher levels of competition as a motivating factor. However, research exploring career development pathways for sport officials remains limited. Future studies may explore this further by examining the FTEM-O pathway (35) to better understand officials' perceptions of different levels within the pathway and their aspirations for advancement. These findings provide valuable insights for organisations seeking to understand retention factors within officiating, highlighting that motivation for officials seems to not be from external reward alone, but by opportunities for growth, identity development, and provide a meaningful contribution within the sporting system.

### Practical implications

The findings of this study offer several practical applications for enhancing sport officials' retention by acknowledging motivational needs associated with their involvement. A key insight is that officials exhibit a stronger intent to continue in the role when their motivational needs are met. Therefore, it is recommended that match official managers engage closely with their officials to understand their motivational profiles. By doing so, organisations will be better positioned to meet the needs of their officiating cohort, potentially improving retention rates.

The results indicate that *Personal Drive and Personal Challenge* are frequently cited motivators, suggesting that match officials are motivated by skill development, knowledge acquisition, and practical experience. To support these intrinsic motivations, development opportunities should be expanded beyond traditional pathways. Match official managers should explore innovative learning and engagement strategies, such as officiating trial matches for representative teams, small-sided games during training sessions, online learning modules, and partnerships with development academies. Additionally, sport governing bodies should consider broadening the scope of educational opportunities beyond formal accreditation programs. Providing structured, accessible learning resources at the national level could reduce duplication of efforts and streamline officiating development pathways (30).

Beyond development opportunities, sport organisations may enhance retention by implementing meaningful reward and recognition strategies that extend beyond financial remuneration, which was the least selected motivator in this study. Any recognition initiative should be co-designed with officials to ensure alignment with their motivational needs, as failure to do so may inadvertently increase attrition. It would be recommended to include officials in organisational decisions, particularly around creating mechanisms to prevent early demotivation and attrition. The findings suggest that officials value autonomy in shaping their career trajectory and seek choice in their development pathways. Given that match official managers often oversee a diverse group across different talent and performance levels, tailored management strategies, particularly informal approaches such as mentoring (30) may be necessary. This is particularly pertinent given the finding that females were more likely to select *Mentor younger officials* compared to males. This provides some context to the literature highlighting that mentoring is a key aspect of the officiating experience for females and the importance placed on mentoring (34, 36). However, this also suggests more education and development may be needed for male officials to engage in and value mentoring, particularly with the role male officials have in providing a supportive and safe culture for females (37).

Motivational profiles vary across performance levels, indicating that developmental programs should adopt a multi-tiered approach, offering distinct support streams for officials at various career stages. For example, development programs for community-level officials, where intrinsic and identified regulation were comparatively lower than elite level officials, may focus on initiatives that enhance perceived competence, social belonging, and clarity of progression. This may also be helpful for younger and less experienced officials, who tend to exhibit poorer levels of mental health than their older and more experienced counterparts (38). This structured approach could enhance role satisfaction and long-term engagement.

Implementing these strategies requires a shift in how match officials are managed, necessitating investment from sport governing bodies. However, such investments are likely to yield positive returns, as research suggests retaining officials is more cost-effective than recruiting and training new ones (6). Given many officials exit the profession within their first five years (6), developing proactive, innovative management strategies could generate long-term benefits. By fostering a supportive and engaging environment, governing bodies can enhance officials' satisfaction, ultimately increasing retention and sustaining a strong officiating workforce.

### Limitations & future research

The findings of this study should be interpreted in light of several limitations. First, while this study provides comparisons across different sports, only four were included, with most classified as interactor sports (39). Future research could adopt a more comprehensive classification, similar to Hancock et al. (8) analysis of interactors, monitors, and reactors. Additionally, although the reasons for continuing officiating were developed in consultation with industry experts, ensuring strong face validity consistent with previous research (8), this study did not utilise a previously validated measurement tool. This limitation should be addressed in future studies through the use or development of validated instruments to assess officiating retention factors more rigorously. Moreover, the use of a convenience sampling approach may have resulted in a sample composed of more intrinsically motivated officials. While we believe this group is broadly representative of the officiating population, the sampling method may have inadvertently attracted more intrinsically motivated officials. To enhance representativeness, future research could collaborate with a single officiating organisation to distribute surveys across its entire network, thereby capturing insights from officials with varying levels of motivation. An additional limitation of the present study is that age was collected using categorical age bands rather than as a continuous variable, which limited the types of statistical analyses that could be conducted, and prevented a more detailed examination of how motivation and intentions may vary across different ages or career stages. Future research should collect age as a continuous variable or consider longitudinal designs to better examine how these factors change across the lifespan and over different stages of officials' careers. Finally, while the quantitative approach used in this study allows for broad comparisons across a diverse officiating population, it does not capture the depth of individual experiences including how in and out group culture impacts on motivation profiles (40). The questionnaire design did not permit examination of when in an officiating career motivational decline occurs, nor the specific experiences that precipitate it. Longitudinal and qualitative research is needed to identify critical career moments and contextual factors that contribute to motivational shifts over time. Future research could incorporate qualitative methodologies, such as in-depth interviews, to provide richer insights into how officials' experiences influence their motivation and retention. The motivation profiles also differed across sports, and as such, future work should be conducted to qualitatively understand how and why these may be sport specific. For example, there may be structures and practices in place specific to the sport that may impact an individual's motivation. Longitudinal studies that track motivation levels in relation to other self-report measures (e.g., commitment, satisfaction, positive mental health, intentions to stay) might help to understand how profiles are predicted by changing motivations in officials' development trajectories. A mixed-methods approach could further enhance understanding by integrating both statistical trends and personal narratives.

## Conclusions

In conclusion, this study makes a valuable contribution to the literature on sports officials' retention, offering important practical implications. Findings indicate that officials are highly motivated by intrinsic and integrated modes of regulation, with no significant gender-based differences. However, key differences emerged based on intention to continue, sport and performance level. *Personal Drive* and *Personal Challenge* were the most commonly cited reasons for continuing in officiating, while *Money* was the least selected. This reinforces the notion that continuing officiating appears to be individually motivated, not financial in nature. Several notable differences in reasons for continuing were observed among performance levels and between different sports. These findings provide valuable insights for governing bodies and officiating managers seeking to improve retention strategies, addressing an ongoing challenge within the profession and need for more effective sport management practices.

## Data Availability

The raw data supporting the conclusions of this article will be made available by the authors, without undue reservation.
